# BMI and perceived weight on suicide attempts in Korean adolescents: findings from the Korea Youth Risk Behavior Survey (KYRBS) 2020 to 2021

**DOI:** 10.1186/s12889-023-16058-z

**Published:** 2023-06-08

**Authors:** Byungmi Kim, Hyo-Seon Kim, Sunhee Park, Jeoung A Kwon

**Affiliations:** 1 National Cancer Control Institute, National Cancer Center, Goyang, 10408 Republic of Korea; 2 National Cancer Center Graduate School of Cancer Science and Policy, Goyang, 10408 Republic of Korea; 3 Public Health at Graduate School, The Catholic University of Korea, Seoul, 06591 Republic of Korea; 4 Institute of Health Services Research, Yonsei University, 50 Yonsei-Ro, Seodaemun-Gu, 03722 Seoul, Republic of Korea

**Keywords:** Adolescent, Suicide attempt, Perceived weight, Underweight, Overweight, Body mass index

## Abstract

**Background:**

Suicide is a leading cause of death in South Korea (hereafter ‘Korea’), and there is evidence that body weight and perceived weight affecting suicide have a significant effect on suicidal behavior in adolescence. This study investigated the association between body mass index (BMI), perceived weight, and suicide attempts in adolescents.

**Methods:**

We included nationally representative data for a total of 106,320 students in our final analysis. We calculated and stratified BMI (underweight, normal weight, overweight) to determine the correlation between BMI and suicide attempts. We stratified the participants into three groups (perceived as underweight, normal weight, and overweight) for subjective body weight perception to analyze the relationship between subjective body weight perception and suicide attempts. We further analyzed the combination of BMI and subjective body weight perception to determine the relationship between suicide attempts and distorted subjective weight perception.

**Results:**

Compared with perceiving oneself as having a normal weight, the odds ratios (ORs) for suicide attempts were significantly increased in the group perceiving themselves as overweight. In addition, those who perceived themselves as overweight but were underweight according to their BMI were at significantly increased risk of suicide attempts relative to those who perceived themselves as about the right weight.

**Conclusions:**

There was a significant association with suicide attempts in the underweight and perceived overweight group. This shows the importance of combining BMI and perceived weight when examining the relationship between weight and suicide attempts in adolescents.

## Introduction

The suicide rate in Korea ranks first among Organization for Economic Cooperation and Development (OECD) countries at 25.4 per 100,000 persons [1]. Suicide accounted for 1.3% of global deaths in 2019, and more than 700,000 people die by suicide every year, the World Health Organization (WHO) reports [2]. The suicide rate trend in Korea is growing more serious, and the suicide rate among young people is increasing faster than that of the older adult population [3]. Suicide is the leading cause of death in Korea, especially among those aged 10 to 39 years [3]. The risk factors for suicide include socioeconomic status, demographic factors, urbanicity, general health behaviors, and other environmental factors. Among the various factors that influence suicide, obesity and weight control may have a significant impact on suicidal behavior in adolescence, and studies on their association are ongoing. Particularly, several studies have shown that perceived weight, weight status, body mass index (BMI), and body satisfaction are important risk factors for suicidal behavior in adolescents [4]. Previous studies have also demonstrated that BMI and perceived weight are equally related to suicidal behavior [4–8]. In another study, people who perceived themselves as overweight had a statistically significant suicide attempt, although they were not overweight according to their BMI [4]. Weight is related to mental health and quality of life, and an abnormal or excessive fat in particular can harm mental health and quality of life [9, 10]. Some people may believe that they will be more likely to attempt suicide if they are obese, and some papers claim this as well [11]. Obese individuals may also have poor mental health because obesity is stigmatized [12–14]. However, many studies have found the opposite, showing fewer suicides and suicide attempts in people with obesity [15–24]. There is further evidence that underweight people have a higher risk of suicide and suicide attempts than those of normal weight [15, 16, 19, 21–25]. Previous studies have shown diverse suicide-related outcomes with regard to obesity. Some of the studies were cross-sectional [5, 7], some were based on small samples [6], and some showed different results according to gender [19] or age [5–7].

Many existing studies have examined the relationship between obesity and suicide [4, 15, 16] or perceived weight and suicide [5, 26, 27]. When individuals perceive themselves as obese, they become afraid of negative social sentiment as the stigma of obesity has been socially internalized; therefore, perceived weight is important [28]. These negative psychological effects can cause suffering, which may intensify suicidal thoughts, suicide plans, and even suicide attempts [28]. Therefore, studying the relationship between suicide and obesity according to the BMI and perceived weight is necessary because both may cause negative psychological effects. However, few have examined the association between suicide attempts and the combination of body mass index (BMI) and subjective body weight perception. Therefore, the purpose of this study was to examine this association in adolescents using data from the national population-based Korea Youth Risk Behavior Survey (KYRBS).

## Materials and methods

### Data source and study population

**Fig. 1:**
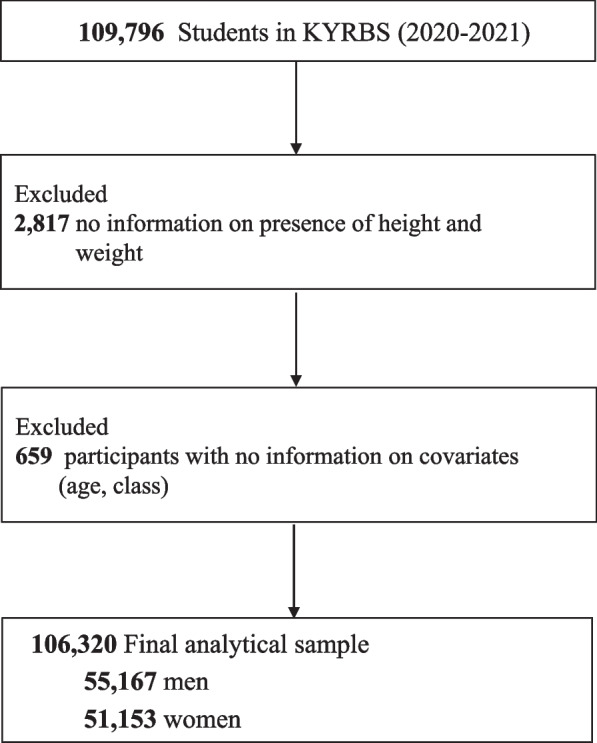
Logistic regression analysis on the association between obesity and suicide attempts flow diagram

This study was based on data collected by the KYRBS from 2020 to 2021. The KYRBS is a cross-sectional survey that has been conducted annually since 2005 by the Korea Centers for Disease Control and Prevention (CDC). The KYRBS is an ongoing national survey that assesses health-risk behaviors among middle- and high-school students, monitors progress toward achieving the national health objectives of Korea’s National Health Plan, and provides data for the development and evaluation of school health policies in South Korea. The KYRBS provides national data that identify the current state and trends in health behaviors of adolescents in Korea, including questions about smoking, drinking, obesity, diet, and physical activity. The data also included information on mental health areas such as obesity status (including perceived weight) and suicide attempts; therefore, this data was selected and investigated in this study. In 2020–2021, 109,796 students (54,948 in 2020 and 54,848 in 2021) from 800 schools (400 middle schools and 400 high schools) were surveyed. This study included 106,320 subjects, excluding those who were not suitable (Fig. 1).

### Body mass index

Anthropometric data, including height and weight, were obtained by the KYRBS, and BMI was calculated by dividing each individual’s weight (kg) by their squared height (m^2^). This study followed the criteria set out in the 2007 Korean Children and Adolescents Standard Growth Chart to classify individuals as underweight (BMI < 5th percentile), normal weight (5th percentile ≤ BMI < 85th percentile), or overweight (BMI ≥ 85th percentile) [29]. In this study, the values calculated according to the adolescent guidelines were BMI < 16.4 for underweight, 16.4 ≤ BMI < 25.5 for normal weight, and BMI ≥ 25.5 for overweight.

### Subjective body weight perception

The survey evaluated self-perceived weight using the topic, “How do you describe your body type?” respondents answered on a scale of 1 to 5 points with a median of 3. This study used a three-point scale consisting of perceived underweight, perceived normal weight, and perceived overweight. Those who responded “very skinny” or “slightly skinny” were measured as perceived underweight, “very fat” or “slightly fat” as perceived overweight, and the midpoint as perceived normal weight.

### Suicide attempts

The KYRBS asked participants about suicide attempts: “Have you attempted suicide in the past 12 months?” Respondents could answer “yes” or “no.” Suicide attempts were coded as “0” for never attempted suicide or “1” for any suicide attempt.

### Data analysis

Because the KYRBS has a structured survey design, the analysis was performed using the SAS PROC SURVEY method, including weights, hierarchical variables, and cluster variables. We used the Student’s t-test for continuous variables and Fisher’s exact test for categorical variables to test the differences in baseline characteristics between students who attempted suicide and those who did not. We used logistic regression to estimate odds ratios (ORs) and 95% confidence intervals (CIs) to compare suicide attempts according to BMI and subjective body weight perception. The analysis was stratified into three groups according to BMI (underweight, normal weight, overweight) and subjective body weight perception status (perceived underweight, perceived normal weight, perceived overweight). Two separate models were evaluated. The first model was adjusted for sex (male or female) and class (middle- or high-school). The second model was further adjusted for sex, class, parental education level (lower than middle school, high school, college, and higher), smoking status (smoker or non-smoker), alcohol consumption (drinker or non-drinker), drug use (yes or no), depressive symptoms (yes or no), and self-perceived overweight (yes or no).

We also investigated the combined effects of BMI and subjective body weight perception on suicide attempts. Combined effects represent the combination of BMI and subjective body weight perception. The following nine groups were defined according to BMI and subjective body weight perception: 1) Underweight/Perceived Underweight, 2) Underweight/Perceived Normal weight, 3) Underweight/Perceived Overweight, 4) Normal weight/Perceived Underweight, 5) Normal weight/Perceived Normal weight, 6) Normal weight/Perceived Overweight, 7) Overweight/Perceived Underweight, 8) Overweight/Perceived Normal weight, 9) Overweight/Perceived Overweight.

All statistical analyses were performed using SAS version 9.4 (SAS Institute, Cary, NC, USA), and *p*-values < 0.05 were considered to indicate statistical significance.

## Results

Table 1 displays the baseline characteristics of all students from KYRBS 2020–2021. The average age of the study subjects was 15.10 ± 1.75 years, and 48.1% were female. Suicide attempts was identified in 2,178 of the 106,320 subjects, representing a 2.0% prevalence of suicide attempts. Compared with students without suicide attempts, those with suicide attempts had depressive symptoms and parents with lower educational attainment, and were more likely to be female, perceive themselves as overweight, non-smokers, drinkers of alcohol, and drug users (All for *p* < 0.0001).

**Table 1:** Characteristics of the study participants (*N* = 106,320)

Characteristics	Total N(%), Mean ± SE	n (%)		*P*-value
		No Suicide attempt	Suicide attempt	
Total	106,320	104,142(98.0)	2,178(2.0)	
Sex				< 0.001
Male	55,167(51.9)	54,423(52.3)	744(34.2)	
Female	51,153(48.1)	49,719(46.8)	1,434(65.8)	
Age	15.1 ± 1,75			
Class				0.004
Middle school	57,611(54.2)	56,350(54.1)	1,261(57.9)	
High school	48,709(45.8)	47,792(45.9)	917(42.1)	
Body mass index (kg/m^2^)				0.023
Underweight (BMI < 5th percentile)	5,330(5.0)	5,195(5.0)	135(6.2)	
Normalweight (5th percentile ≤ BMI < 85th percentile)	85,019(80.0)	83,283(80.0)	1,736(79.7)	
Overweight (BMI ≥ 85th percentile)	15,971(15.0)	15,664(15.0)	307(14.1)	
Subjective body weight perception				< 0.001
Perceived underweight	26,691(25.1)	26,191(25.2)	500(23.0)	
Perceived normalweight	38,808(36.5)	38,094(36.6)	714(32.8)	
Perceived overweight	40,821(38.4)	39,857(38.3)	964(44.3)	
Smoking status				< 0.001
Smokers	10,463(9.8)	9,925(9.5)	538(24.7)	
Non-smoker	95,857(90.2)	94,217(90.5)	1,640(75.3)	
Alcohol Consumption				< 0.001
Drinker	34,952(32.9)	33,794(32.5)	1,158(53.2)	
Non-drinkers	71,368(67.1)	70,348(67.6)	1,020(46.8)	
Drug use				< 0.001
Yes	715(0.7)	606(0.6)	109(5.0)	
No	105,605(99.3)	103,536(99.4)	2,069(95.0)	
Depressive symptom				< 0.001
Yes	27,438(25.8)	25,671(24.7)	1,767(81.1)	
No	78,882(74.2)	78,471(75.4)	411(18.9)	
Educational level of father				< 0.001
Less than middle school	1,146(1.1)	1,084(1.5)	62(4.0)	
High school	17,442(16.4)	17,073(26.9)	369(28.4)	
College and higher	44,502(41.9)	43,657(71.5)	845(67.6)	
Educational level of mother				< 0.001
Less than middle school	892(0.8)	848(1.2)	44(2.9)	
High school	20,578(19.4)	20,136(30.6)	442(32.4)	
College and higher	43,832(41.2)	42,980(68.3)	852(64.8)	

BMI: Underweight (BMI < 16.4), Normalweight (16.4 ≤ BMI < 25.5), Overweight (BMI ≥ 25.5)

Table 2 shows the calculated ORs and 95% CIs for suicide attempts based on BMI and subjective body weight perception. In subgroups stratified by BMI categories, being underweight was significantly associated with increased odds of having a suicide attempt compared with the normal weight group (adjusted OR = 1.31, 95% CI:1.06–1.63) after adjusting for covariates (model 2). In subgroups stratified by subjective perception of body weight categories, compared with those who perceived themselves as having a normal weight, the ORs for suicide attempts were significantly increased in those who perceived themselves as underweight (adjusted OR = 1.14, 95% CI:1.00–1.29) and overweight (adjusted OR = 1.31, 95% CI:1.18–1.45) (Model 1). After adjusting for covariates in Model 2, these findings were significant in the perceived overweight group (adjusted OR = 1.16, 95% CI: 1.04–1.29) but not in the perceived underweight group.

**Table 2:** The relationship of suicide attempt according to BMI and Subjective body weight perception status

Characteristics	OR(95% CI)	
	Model 1	Model 2
Body mass index (kg/m^2^)^a^		
Underweight (BMI < 5th percentile)	1.18 (0.96–1.46)	1.31 (1.06–1.63)
Normalweight (5th percentile ≤ BMI < 85th percentile)	1.00	1.00
Overweight (BMI ≥ 85th percentile)	1.07 (0.93–1.23)	0.92 (0.78–1.09)
Subjective body weight perception^b^		
Perceived underweight	1.14 (1.00–1.29)	1.04 (0.91–1.18)
Perceived normalweight	1.00	1.00
Perceived overweight	1.31 (1.18–1.45)	1.16 (1.04–1.29)

*OR* Odds ratio, *CI* Confidence interval

^a^Model 1 is adjusted for sex, class. Model 2 is adjusted for sex, class, alcohol consumption, smoking, drug use, self-perceived overweight, educational level(father, mother), depressive symptom

^b^Model 1 is adjusted for sex, class. Model 2 is adjusted for sex, class, alcohol consumption, smoking, drug use, educational level(father, mother), depressive symptom

Table 3 shows the association between suicide attempts and the combination of obesity status and subjective body weight perception. Using “about the right weight” as a reference, underweight students were more likely to perceive themselves as underweight, normal-weight students were more likely to perceive themselves as having a normal weight, and overweight students were more likely to perceive themselves as overweight. The proportions of students who correctly perceived their body weight status were 89.8% in the underweight group, 44.3% in the normal weight group, and 95.6% in the overweight group. We observed that those who perceived themselves as overweight but were underweight or a normal weight according to their BMI were at greater risk of suicide attempts than students who perceived themselves as approximately the right weight. In contrast, students who were overweight according to their BMI had a lower risk of suicide attempts than those who correctly perceived their weight, although the difference was not statistically significant. In particular, those who perceived themselves as overweight but were underweight according to their BMI were at significantly increased risk of suicide attempts (adjusted OR = 5.95, 95% CI: 1.12–31.75) relative to those who perceived themselves as approximately the right weight.

**Table 3:** The association of suicide attempt according to the combination of BMI and Subjective body weight perception

Variables	N(%)	Suicide attempt			
		Model 1		Model2	
		*OR*adj (95% *CI*)	*p*-value	*OR*adj (95% *CI*)	*p*-value
Underweight					
Underweight/Perceived Underweight	4,810 (89.8)	1.00	-	1.00	-
Underweight/Perceived Normalweight	469 (8.8)	1.17 (0.41–3.33)	0.770	1.55 (0.53–4.50)	0.423
Underweight/Perceived Overweight	77 (1.4)	2.97 (0.69–12.86)	0.145	5.95 (1.12–31.75)	0.037
Normalweight					
Normalweight/Perceived Underweight	22,001 (25.7)	1.06 (0.87–1.29)	0.544	0.97 (0.79–1.18)	0.729
Normalweight/Perceived Normalweight	27,944 (44.3)	1.00	-	1.00	-
Normalweight/Perceived Overweight	25,630 (30.0)	1.33 (1.14–1.56)	< 0.001	1.14 (0.97–1.34)	0.121
Overweight					
Overweight/Perceived Underweight	57 (0.4)	0.51 (0.06–4.04)	0.521	0.53 (0.05–5.23)	0.582
Overweight/Perceived Normalweight	647 (4.0)	0.67 (0.20–2.26)	0.521	0.61 (0.18–2.04)	0.420
Overweight/Perceived Overweight	15,344 (95.6)	1.00	-	1.00	-

*OR* odds ratio, *CI* confidence interval

Model 1 is adjusted for sex, class

Model 2 is adjusted for sex, class, alcohol consumption, smoking, drug use, self-perceived overweight, educational level(father, mother), depressive symptom

## Discussion

This study investigated the association between suicide attempts and the combination of BMI and subjective body weight perception in Korean adolescents using nationwide KYRBS data. Regarding the BMI and suicide attempts, the underweight group tended to make more suicide attempts than the normal weight group; regarding the subjective body weight perception and suicide attempts, the overweight group tended to make more suicide attempts than the normal weight group.

Regarding the combination of BMI and subjective body weight perception, individuals who were underweight and perceived themselves as overweight tended to make more suicide attempts than individuals who were underweight and perceived themselves as underweight. The results were adjusted for demographic variables, including age, sex, alcohol consumption, smoking, drug use, weight self-perception, parents’ educational level, and depressive symptoms.

Previous studies have indicated a relationship between obesity status and mental health, but the direction of the results varies. In one cohort study, those who were underweight showed a lower tendency for suicide ideation [15, 17]. A study of the US National Health Interview Survey indicated a reversed association between body weight and suicide [16]. A cohort study of Swedish men similarly showed that suicide attempts decreased as BMI increased [19, 24]. In the Korean Cancer Prevention Study, those with biological cardiovascular disease risk factors, including being underweight, tended to have a higher suicide mortality rate [21]. The Taiwanese and British adults cohort study and the Veterans Affairs health system in the US have shown similar results [11, 22, 23, 25].

Several studies have produced results similar to those of our study regarding self-perception of being overweight and suicidal ideation [5]. The risk of suicidal ideation, suicide plans, or suicide attempts, which define suicidality, related to the self-perception of being overweight increased from 5.7 percentage points in 1999–2001 to 10.1 points in 2015–2017 according to the Youth Risk Behavior Survey among US adolescents in grades 9–12 [28]. Furthermore, in a systematic review and meta-analysis, those with a self-perception of being overweight tended to have poor mental health, including depression, attempted suicide, depressive symptoms, and suicidal ideation [30].

The US Youth Risk Behavior Survey presented a similar relationship between suicide attempts and the combination of obesity status and subjective body weight perception [4]. The results showed that those with a self-perception of being overweight while not being overweight tended to have more suicide attempts [4]. These results support the fact that a statistically significant number of suicide attempts were made when there was a difference between individuals’ actual and perceived weight, such as when a person perceived that they were overweight despite being underweight as in our thesis results [4, 31].

Furthermore, adolescent suicide attempts may be influenced by the gap between socially constructed body image—which can result from social norms, stigma, and influence—and the definition and reality of obesity [4, 31]. Self-perceived abnormal weight (i.e., overweight or underweight) in adolescence is strongly associated with negative psychiatric conditions, such as suicidal behavior [26, 27, 32–35]. These results show the causal relationship between self-perceived weight and suicidal behavior [5].

This study had several limitations. First, it followed a cross-sectional design; therefore, it is difficult to confirm a causal relationship. Moreover, reporting biases are possible because respondents’ height and weight were self-reported data. Second, our study had some reliability limitations in our results. Our results showed a wide confidence interval owing to the small sample size. Finally, this study only included students who were attending school, so it does not reflect the results of adolescents who did not attend school.

Despite these limitations, this study presents a new perspective by examining the relationship between adolescent BMI and suicide attempts alone and in combination with self-perceived weight from Korean national data. Apart from one’s actual and perceived weight, one’s desire to lose weight will be an important variable in understanding suicide attempts in future research.

## Conclusion

This study showed the relationship between suicide attempts and the combination of BMI and subjective body weight perception among Korean adolescents using data from the nationwide KYRBS. There was a significant relationship between the underweight/perceived-overweight group and suicide attempts. This study provides a new perspective on the relationship between weight and suicide attempts in adolescents by combining BMI and self-perceived weight.

## Data Availability

Publicly available datasets were analyzed in this study. These data can be found at https://www.kdca.go.kr/yhs/.
